# Habitual Consumption of Soy Products and Risk of Nasopharyngeal Carcinoma in Chinese Adults: A Case-Control Study

**DOI:** 10.1371/journal.pone.0077822

**Published:** 2013-10-14

**Authors:** Yuan-ting Liu, Yu-ying Fan, Chun-hua Xu, Xiao-ling Lin, Yun-kai Lu, Xing-lan Zhang, Cai-xia Zhang, Yu-ming Chen

**Affiliations:** 1 Guangdong Provincial Key Laboratory of Food, Nutrition and Health, School of Public Health, Sun Yat-sen University, Guangzhou, People's Republic of China; 2 Sun Yat-sen University Cancer Center, Guangzhou, People’s Republic of China; 3 Sun Yat-sen University Ophthalmic Center, Guangzhou, People’s Republic of China; 4 Central Hospital of Panyu District, Guangzhou, People’s Republic of China; Health Canada and University of Ottawa, Canada

## Abstract

**Background and Objectives:**

Many studies have shown a negative association between the consumption of soy products and the risk of some cancers, but little is known about the effect of soy consumption on nasopharyngeal carcinoma. We assessed the association between the consumption of soy products on nasopharyngeal carcinoma risk in Chinese individuals.

**Methods:**

This case-control study included 600 (448 males and 152 females) incident cases of nasopharyngeal carcinoma, and an equal number of controls, matched according to gender, age (± 3 y) and household type to the nasopharyngeal carcinoma cases. All subjects were recruited from hospitals in Guangzhou, China. A face-to-face interview was conducted with each study individual to collect general information and habitual dietary intake using a 78-item quantitative food-frequency questionnaire. Odds ratios and their 95% confidence intervals were estimated using conditional logistic regression analyses.

**Results:**

The median intakes of soy foods (in protein) were 0.5/0.5, 1.4/1.7, 2.7/3.3 and 6.1/7.7 (male/female) g/d in the quartiles 1 to 4. Both univariate and multivariate analyses showed no significant association between the consumption of soy proteins or soy isoflavones and the risk of nasopharyngeal carcinoma. The adjusted odds ratios (95% confidence intervals) between extreme quartiles were 0.97 (0.66-1.45) for soy proteins and 0.97 (0.66-1.42) for total isoflavones. Null associations were also observed between intake of the individual isoflavones daidzein, genistein and glycitein and NPC risk, with adjusted odds ratios for the extreme quartiles ranging between 0.73 and 1.23.

**Conclusion:**

Habitual consumption of soy products had no significant effect on the risk of nasopharyngeal carcinoma in Chinese adults with a relatively low intake.

## Introduction

South China has a much higher incidence of nasopharyngeal carcinoma (NPC, 20 per 10^5^ males and 7 per 10^5^ females per year) compared with the rest of the world (1.7 per 10^5^ persons per year) [1]. However, the incidence of NPC is lower among Chinese migrants living in low-risk areas, which suggests that environmental factors (for instance, dietary factors) may contribute to the diversity of NPC risk [2]. Cantonese-style salted fish is widely accepted as a causative environmental risk factor for NPC [3], but little is known of the association between the risk of NPC and other dietary factors such as soy products.

Epidemiological evidence has demonstrated the favorable effect of soy products on breast [4,5], prostate [6], lung [7] and colorectal cancer [8]. In vitro studies showed that the bioactive compounds of soy products, dietary isoflavones, conferred inhibitory effects on NPC cells [9,10]. Therefore, we hypothesized that soy products may play a protective role in NPC carcinogenesis. In the limited number of published studies that investigated a few specific soy products (tofu [11] and fermented soybean products [7]), no association was found between the intake of soy products and the risk of developing NPC. However, these previous studies were limited due to their small sample size (<500 incident cases), and the fact that they did not sufficiently control for potential confounders (such as socioeconomic status and total energy intake, among other factors). Thus, additional studies are required to clarify the association between soy consumption and NPC risk. Given that Cantonese people are susceptible to NPC and that the Cantonese diet is soy-rich, we performed a case-control study in Guangdong to assess the association between the consumption of soy products and the risk of developing NPC.

## Materials and Methods

### Study population

We conducted this 1:1 matched, case-control study between July 2009 and March 2011 in Guangzhou, China. The details of the methods used have been reported previously [12]. In brief, eligible cases were in-patients at the *Sun Yat-sen University Cancer Center*, were aged between 32 and 73 years and had histological diagnoses of NPC up to 3 months prior to interviews. The participants were restricted to inhabitants who had lived in the Guangdong Province for at least ten years. Exclusion criteria for cases included the following: (1) significant self-reported dietary changes over the past five years; (2) a family history of NPC within 3 generations; (3) evidence of chronic diseases which might change their dietary habits (e.g., diabetes, stoke, coronary disease, malignant cancer); (4) language difficulties that hindered the interviews. The control subjects, who met similar inclusion and exclusion criteria as the cases, with the exception of NPC, cataract or poor vision diagnosis, were selected from the *Sun Yat-sen University Ophthalmic Center*. They were matched to cases by gender, age (±3 y) and household type in a ratio of 1:1. The study areas in Guangdong province for the cases and controls were comparable. The study protocol was approved by the Ethics Committee of the School of Public Health at Sun Yat-sen University. Written informed consent was obtained from all of the participants at the enrollment.

### Data collection

Subjects in our study were interviewed face-to-face using a standardized and structured questionnaire, which collected information about demographic characteristics, lifestyle factors, habitual dietary intake in the year preceding the survey and other potential NPC risk factors. Additional information about cancer stage groups TNM (tumor-node-metastasis) [13], histological types and VCA/EA-IgA (Viral capsid antigen/Early antigen-antibody A) antibody titers was obtained from the medical records of the cases. The TNM stages were classified into four categories according to the 6th edition of the International Union against Cancer/American Joint Committee on Cancer (UICC/AJCC) staging system [13]. The proportion of cases and controls interviewed by each interviewer was similar.

Habitual dietary consumption was evaluated using a 78-food-item quantitative food-frequency questionnaire (FFQ), with moderately high validity in the source population (correlation coefficients were 0.45 for soy and 0.46-0.65 for macronutrients) [14]. The average consumption of soy products (per year, month, week or day) was collected using color photographs of different foods and portion sizes as visual aids. We examined the intake of 7 specific soy products, including firm tofu, soft tofu, bean curd skin (e.g., vegetarian duck, tofu stick, and bean curd sheet), soy milk, dried soybeans and soybean sprouts. In our study, habitual consumption of soy products had satisfactory short-term reproducibility, with correlation coefficients of 0.569 for total isoflavones and 0.647 for soy proteins (n=32).

Daily intake of soy proteins, which was used to measure the total combined consumption of all soy products, was calculated by using the 2004 China Food Composition Table [15], as well as daily energy intake and other food groups (such as cereals, dairy products, vegetables and fruits). We estimated the habitual consumption of total isoflavones and its major components (daidzein, genistein and glycitein) on the basis of a food composition table of soy products that was specially developed in Hong Kong [16].

### Data analysis

Statistical analyses were performed using SPSS 13.0 (SPSS Inc., Chicago, Illinois, USA). The intake of both soy proteins and isoflavones was log-transformed (natural log) to obtain normal distributions. To minimize the confounding effect of energy, we computed the energy-adjusted intake of both the soy proteins and the isoflavones as the residuals from the regression models separately [17], and then categorized these residuals into gender-specific quartiles according to the distribution of the controls. The associations between NPC risk and energy-adjusted intake of soy proteins and isoflavones were measured using odds ratios (OR) and corresponding 95% confidence intervals (95%CI) in conditional logistic regression models (p values quoted are 2-sided). Categories of dietary exposure were considered as ordinal variables in the test for trends. Using stepwise methods in the logistic regression models, adjustments were made for potential confounding factors (such as marital status, occupational and domestic exposure to toxic substances, rhinitis history, education level, occupation, household income, area of residence, smoking status, alcohol consumption, physical activity, body mass index (BMI), and intake of daily energy and other food groups (g/d, continuous)). To examine the potential effect modification of the disease exposure associations by gender, household type, TNM stage, education level, dietary intakes of vegetables or fruits , stratified analyses were conducted, and interaction terms were tested. We performed additional sensitivity analyses that were restricted to case-control pairs with and without matching educational levels.

Power analysis of cases and controls in the highest and lowest quartiles (Q4 and Q1) showed that we would have over 80% power to detect an OR≤ 0.725 (or ≥ 1.300) for the consumption of soy proteins and isoflavones at a significance level of 0.05 (two-tailed).

## Results

In our study, the exclusion criteria of total eligible cases (n=653) included implausible values for total energy intake (<700 or >4200 kcal for men, <500 or >3500 kcal for women, n=2), subjects who were uncomfortable with completing the questionnaire (n=20) or who refused to participate (n=31); we were left with 600 case-control pairs as a consequence. Of the 600 cases, 510 had late stage (III & IV) cancer. A majority of cases were diagnosed as undifferentiated carcinoma (95%) and were seropositive for EBV VCA-IgA (93%) and EA-IgA (75%).

There was no significant difference in cases and controls regarding age, gender and household type, all of which were matched during recruitment. Table 1 displays the distributions of demographic and lifestyle characteristics and selected NPC risk factors in cases and controls. Male cases tended to have higher body mass index (BMI), be better educated and have occupations that were less active compared with the controls. Male cases were also more likely to have a history of chronic rhinitis and passive smoking and to have been exposed to heat and toxic substances such as organic solvents, incense, new furniture and interior decorating than controls. Female cases had a higher income and more exposure to new furniture and house decorations than controls.

**Table 1:** Demographic and lifestyle characteristics and selected NPC risk factors of the study population in Guangzhou, China ^1^ .

	**Males**					**Females**			
	Cases (n=448)	Controls (n=448)	OR (95%CI)	P		Cases (n=152)	Controls (n=152)	OR (95%CI)	P
Age (*y*)	47.9±9.1	47.7±9.1	1.00 (0.99-1.02)	0.816		45.9±8.7	46.3±8.7	0.99 (0.97-1.02)	0.690
Marital status, *n*(*%*)									
Married	443(98.9)	431(96.2)	3.40 (1.25-9.22)	0.016		147(96.7)	143(94.1)	2.33 (0.60-9.02)	0.220
Unmarried/Divorced/Widowed	5(1.1)	17(3.8)	1.00			5(3.3)	9(5.9)	1.00	
Household type, *n* (*%*)				1.000					1.000
Urban	299(66.7)	299(66.7)	--			100(65.8)	100(65.8)	--	
Rural	149(33.3)	149(33.3)	--			52(34.2)	52(34.2)	--	
Educational level, *n*(*%*)									
Primary school or below	70(15.6)	92(20.5)	1.00			39(25.7)	38(25.0)	1.00	
Secondary school	145(32.4)	177(39.5)	1.13 (0.77-1.67)	0.535		45(29.6)	52(34.2)	0.85 (0.43-1.65)	0.623
High school	136(30.4)	123(27.5)	1.68 (1.10-2.57)	0.17		34(22.4)	35(23.0)	1.01 (0.49-2.08)	0.983
College or above	97(21.7)	56(12.5)	3.02 (1.78-5.10)	<0.001		34(22.4)	27(17.8)	1.25 (0.58-2.70)	0.570
Occupation ^3^, *n*(*%*)									
Light intensity	171(38.2)	148(33.0)	1.00			57(37.5)	44(28.9)	1.00	
Moderate intensity	141(31.5)	113(25.2)	1.06 (0.75-1.51)	0.311		47(30.9)	45(29.6)	0.74 (0.42-1.32)	0.311
Heavy intensity	136(30.4)	187(41.7)	0.56 (0.39-0.79)	0.001		48(31.6)	63(41.4)	0.49 (0.26-0.93)	0.029
Household income, *yuan/ month/ person*, *n*(*%*)									
≤ 500	39(8.7)	47(10.5)	1.00			15(9.9)	21(13.8)	1.00	
501~1500	160(35.7)	180(40.2)	1.13 (0.69-1.86)	0.617		42(27.6)	56(36.8)	1.22 (0.52-2.84)	0.652
1501~3000	109(24.3)	106(23.7)	1.44 (0.82-2.50)	0.202		49(32.2)	47(30.9)	1.54 (0.65-3.65)	0.324
>3000	140(31.3)	115(25.7)	1.72 (0.99-3.00)	0.056		46(30.3)	28(18.4)	2.34 (0.96-5.66)	0.060
Occupational exposure to toxic substances									
Heat ^2^, *n*(*%*)	25(5.6)	12(2.7)	2.08 (1.05-4.15)	0.037		7(4.6)	3(2.0)	2.33 (0.60-9.02)	0.220
Organic solvent ^2^, *n*(*%*)	54(12.1)	31(6.9)	1.79 (1.14-2.82)	0.012		14(9.2)	10(6.6)	1.50 (0.61-3.67)	0.374
Domestic exposure to potential toxic substances									
House decoration ^2^, *n*(*%*)	165(36.8)	101(22.5)	1.97 (1.47-2.65)	<0.001		62(40.8)	39(25.7)	2.10 (1.25-3.52)	0.005
New furniture ^2^, *n*(*%*)	155(34.6)	94(21.0)	2.00 (1.47-2.72)	<0.001		51(33.6)	32(21.1)	1.79 (1.09-2.95)	0.014
Incense ^2^, *n*(*%*)	227(50.7)	172(38.4)	1.69 (1.28-2.23)	<0.001		76(50.0)	62(40.8)	1.44 (0.92-2.26)	0.115
Chronic rhinitis history ^2^, *n*(*%*)	130(29.0)	76(17.0)	1.98 (1.43-2.74)	<0.001		34(22.4)	32(21.1)	1.08 (0.63-1.86)	0.781
Smoking status ^4^, *n*(*%*)									
Smoker	220(49.1)	213(47.5)	1.00			1(0.7)	0 (0.0)	--	
Ex-smoker	72(16.1)	63(14.1)	1.25 (0.84-1.86)	0.276		1(0.7)	1(0.7)	--	
Nonsmoker	156(34.8)	172(38.4)	1.13 (0.85-1.50)	0.408		150(98.7)	151(99.3)	--	
Alcohol consumption, *n*(*%*)	128(28.6)	116(25.9)	1.15 (0.85-1.54)	0.366		4(2.6)	5(3.3)	0.80 (0.22-2.98)	0.739
Passive smoking ^2,5^, *n*(*%*)	277(61.8)	234(52.2)	1.57 (1.18-2.10)	0.002		72(47.4)	58(38.2)	1.50 (0.93-2.42)	0.097
Physical activity ^6^ (*MET∙h /d*)	38.6±9.7	39.8±12.1	0.99 (0.98-1.00)	0.094		37.8±8.5	38.6±11.0	0.99 (0.96-1.02)	0.396
Body mass index (*kg/m* ^*2*^)	23.5±3.0	22.8±2.8	1.08 (1.03-1.13)	0.001		22.5±3.2	22.4±2.8	1.01 (0.94-1.09)	0.778
Dietary energy intake, *kcal/d*	1997±585	2081±644	0.9998 (0.9995- 0.9999)	0.040		1507±482	1576±467	1.000(0.999-1.000)	0.205

^1^ Continuous variables were shown as the means±standard deviation. Categorical variables were shown as numbers (percents). Variables were evaluated in conditional logistic model.

^2^ Variables were scored as yes or no, and numbers (percents) of yes responses were listed. House decoration refers to replacement of floor board, wall, and indoor painting for almost a whole flat or house; new furniture refers to a set of new furniture for living room(s) and bed room(s).

^3^ Occupation was categorized into 3 levels on the basis of the intensity of occupational physical activity.

^4^ Smoking status was defined as current smoker, ex-smoker or nonsmoker (a smoker was defined as someone smoking at least one cigarette per day for at least six months).

^5^ Passive smoking was designated as exposure to other peoples' tobacco smoke for at least 5 minutes daily in the previous five years.

^6^ Physical activity was defined as occupational, leisure time and household chores activity, and evaluated by metabolic equivalent hours per day (MET∙ h/d).

Abbreviations: BMI: body mass index; NPC: Nasopharyngeal carcinoma; MET: metabolic equivalent

The estimated energy-adjusted intake of soy proteins ranged from 0.05 to 25.1 g/d, with a mean of 2.68 g/d in males and 3.29 g/d in females, while the daily intake of isoflavones ranged from 0.09 mg to 52.8 mg, with a mean of 6.84 mg and 8.34 mg in males and females, respectively. The main sources of soy products were soft tofu, soybean sprouts and soy milk; these products constituted 60% of the total soy protein intake (data not shown).

Table 2 shows the association between soy and isoflavones and the risk of NPC. In the univariate conditional logistic regression analyses, ORs (95% CI) comparing extreme quartiles were 0.76 (0.54-1.06) for soy proteins (p=0.130) and 0.75 (0.55-1.03) for total isoflavones (p=0.106). The corresponding multivariate-adjusted ORs (95% CIs) were 0.97 (0.66-1.45) for soy proteins and 0.97 (0.66-1.42) for total isoflavones. No significant associations were found between any of the three isoflavone components (daidzein, genistein and glycitein) and NPC risk.

**Table 2:** Association between soy and isoflavone intake and NPC in Guangzhou, China.

	Quartiles of dietary energy-adjusted intake				
Variable	1	2	3	4 (highest)	P-trend
Soy proteins					
Median intake (M/F), *g/d*	0.52/0.54	1.37/1.67	2.72/3.28	6.11/7.66	
N Cases/ Controls	154/149	165/150	161/148	118/150	
OR I (95%CI)	1.00	1.04(0.76-1.44)	1.04(0.75-1.43)	0.76(0.54-1.06)	0.130
OR II (95%CI)	1.00	0.90(0.62-1.30)	1.15(0.78-1.68)	0.97(0.66-1.45)	0.797
Total isoflavones					
Median intake (M/F), *g/d*	1.66/1.42	4.51/4.39	8.12/8.19	16.3/19.4	
N, Cases/ Controls	172/149	146/150	150/148	130/150	
OR I (95%CI)	1.00	0.83(0.61-1.15)	0.87(0.63-1.19)	0.75(0.55-1.03)	0.106
OR II (95%CI)	1.00	0.74(0.51-1.08)	0.95(0.65-1.14)	0.97(0.66-1.42)	0.842
Daidzeins					
Median intake (M/F), *g/d*	0.54/0.57	1.77/1.74	3.18/3.24	6.54/7.73	
N, Cases/ Controls	168/149	148/150	152/148	130/150	
OR I (95%CI)	1.00	0.93(0.68-1.27)	0.85(0.61-1.17)	0.80(0.58-1.10)	0.926
OR II (95%CI)	1.00	0.80(0.55-1.16)	1.02(0.69-1.51)	1.04(0.71-1.52)	0.609
Genisteins					
Median intake (M/F), *g/d*	0.72/0.75	1.86/2.33	3.60/4.32	8.28/10.1	
N Cases/Controls	173/149	143/150	154/148	128/150	
OR I (95%CI)	1.00	0.82(0.60-1.12)	0.89(0.65-1.23)	0.74(0.54-1.02)	0.101
OR II (95%CI)	1.00	0.73(0.51-1.06)	1.01(0.69-1.50)	0.94(0.64-1.37)	0.901
Glyciteins					
Median intake (M/F), *g/d*	0.08/0.08	0.24/0.31	0.51/0.64	1.28/1.52	
N Cases/Controls	146/149	181/150	133/148	138/150	
OR I (95%CI)	1.00	1.22(0.90-1.67)	0.90(0.64-1.25)	0.93(0.67-1.30)	0.324
OR II (95%CI)	1.00	1.10(0.76-1.59)	0.97(0.66-1.43)	1.23(0.83-1.83)	0.433

OR I and OR II: odds ratios from conditional logistic model, OR I: without further adjustment; OR II: adjusted for BMI, education level, marital status, occupation, household income, occupational and domestic exposure to potential toxic substances, chronic rhinitis history, smoking status, passive smoking, daily energy intake (log-transformed) and energy-adjusted intake of other food groups (including preserved vegetables, cereals, fresh meats, preserved meats, roasted meats, dairy products, nuts, vegetables and fruits) by a stepwise forward method.95%CI: 95% confidence interval.

Abbreviations: see Table 1.

We evaluated the association between the risk of NPC and intake of soy proteins and isoflavones separately across different subgroups stratified by gender, household type, TNM stage group, education level and vegetable or fruit intake. In the subgroup of males, the adjusted OR for the second quartile of total isoflavones consumption was 0.59 (95% CI: 0.37–0.94) in male. None of the ORs reached statistical significance in any of the analyses in females or across subgroups of household types, TNM stage or matched educational level; vegetable or fruit intake.There was no evidence of effect modification by factors evaluated in the subgroup analysis, with p-values for interactions ranging from 0.669 to 0.998. ORs from subsequent analyses of the 358 and 242 case-control pairs with matched and unmatched educational levels, respectively, were generally similar to those from the whole data set. (Table 3)

**Table 3:** Association between the intake of soy products and isoflavones and NPC by gender, household type, TNM stage, and educational level in Guangzhou, China ^1^.

	Quartiles of dietary energy-adjusted intake				P-trend	P for
Variable	1	2	3	4 (highest)		interaction
***Soyproteins***						
Gender						0.669
Male	1.00	0.78(0.50-1.21)	1.15 (0.73-1.81)	0.97(0.61-1.53)	0.750	
Female	1.00	1.18(0.55-2.58)	1.32(0.62-2.84)	1.06(0.47-2.38)	0.821	
Household type						0.760
Urban	1.00	1.17(0.69-1.98)	1.32(0.77-2.25)	1.22(0.69-2.15)	0.434	
Rural	1.00	0.60(0.28-1.27)	0.94(0.44-2.00)	0.63(0.29-1.38)	0.411	
TNM stage						0.801
I & II	1.00	0.50(0.20-1.21)	0.74(0.29-1.91)	0.70(0.29-1.66)	0.579	
III&IV	1.00	0.97(0.64-1.46)	1.23(0.81-1.87)	1.01(0.65-1.56)	0.691	
Matched educational level^2^						0.813
<High school	1.00	1.01(0.57-1.80)	1.74(0.86-3.51)	0.76(0.37-1.56)	0.982	
≥High school	1.00	0.81(0.35-1.88)	0.75(0.33-1.67)	0.70(0.30-1.66)	0.439	
Educational level^2^						0.842
Matched	1.00	0.94(0.59-1.51)	1.18(0.73-1.92)	0.90(0.55-1.48)	0.932	
Unmatched	1.00	0.76(0.42-1.40)	1.06(0.56-2.00)	0.84(0.44-1.60)	0.832	
Vegetables intake						0.222
≤ Median	1.00	0.89(0.47-1.69)	1.04(0.53-2.02)	1.28(0.65-2.50)	0.439	
> Median	1.00	0.57(0.24-1.33)	0.46(0.17-1.22)	0.54(0.22-1.36)	0.207	
Fruit intake						0.905
≤ Median	1.00	0.86(0.43-1.73)	0.69(0.34-1.40)	0.74(0.36-1.53)	0.316	
> Median	1.00	1.30(0.57-2.96)	0.97(0.41-2.32)	1.36(0.55-3.39)	0.651	
***Total isoflavones***						
Gender						0.975
Male	1.00	0.59(0.37-0.94)	0.99(0.63-1.55)	0.89(0.56-1.39)	0.967	
Female	1.00	1.21(0.57-2.59)	0.88(0.37-2.08)	1.37(0.63-2.97)	0. 528	
Household type						0.853
Urban	1.00	1.09(0.63-1.87)	0.95(0.55-1.62)	1.16(0.67-2.01)	0.691	
Rural	1.00	0.65(0.31-1.35)	0.89(0.42-1.89)	0.72(0.34-1.52)	0.443	
TNM stage						0.692
I & II	1.00	0.43(0.17-1.04)	0.73(0.29-1.85)	0.73(0.30-1.71)	0.753	
III&IV	1.00	0.84(0.55-1.28)	1.01(0.66-1.55)	1.02(0.68-1.56)	0.731	
Matched educational level^2^						0.738
<High school	1.00	0.72(0.38-1.33)	1.56(0.82-2.99)	0.82(0.42-1.59)	0.891	
≥High school	1.00	0.58(0.25-1.33)	0.74(0.34-1.63)	0.74(0.32-1.73)	0.916	
Educational level^2^						0.944
Matched	1.00	0.66(0.41-1.06)	0.94(0.58-1.52)	0.85(0.53-1.39)	0.883	
Unmatched	1.00	0.78(0.41-1.45)	0.88(0.46-1.68)	0.95(0.51-1.78)	0.953	
Vegetables intake						0.205
≤ Median	1.00	0.68(0.35-1.32)	0.99(0.51-1.90)	1.43(0.73-2.82)	0.240	
> Median	1.00	0.64(0.27-1.54)	0.68(0.26-1.79)	0.49(0.20-1.23)	0.155	
Fruit intake						0.998
≤ Median	1.00	0.88(0.46-1.71)	0.77(0.36-1.63)	0.85(0.42-1.73)	0.570	
> Median	1.00	1.31(0.55-3.11)	1.10(0.46-2.64)	1.30(0.55-3.07)	0.697	

^1^ Values were odds ratios (95% CIs) from multivariate models (see OR II in Table 2),

^2^ 358 pairs matched for educational level and 242 pairs unmatched for education level.

Abbreviations: NPC: Nasopharyngeal carcinoma; OR: odds ratio; CI: confidence interval; TNM: International Union Against Cancer/American Joint Committee on Cancer staging system for tumor-node-metastasis.

## Discussion

In this matched case-control study with 600 pairs of incident NPC cases and controls, we found no significant protective effects of soy proteins or total or individual soy isoflavones on NPC, either overall or across groups of gender, household type, TNM stage and education level.

Although a large number of epidemiological studies observed favorable effects of soy products on breast [7], prostate [6], lung [18] and colorectal cancer [8], few studies have examined the association between soy products and NPC risk, and the results in these latter studies are conflicting. A case-control study of 103 pairs matched in a ratio of 1:2 in Yangjiang, China, found that consumption of tofu was associated, with an OR of 2.36 (95% CI 0.26-21.27), with NPC risk. Other studies mainly focused on the relationship between fermented soy products and NPC risk. A hospital-based study of 128 cases and 174 controls in Guangxi province (in southwestern China) reported a significant trend (p<0.01), but a non-significant increase, in NPC risk related to fermented soy products [19]. In contrast, a population-based study in Taiwan (using 375 cases and 327 controls) showed a non-significant decreased risk of NPC associated with the intake of fermented soybean products in early age [20]. No significant association was observed between NPC risk and fermented bean curd intake among Singapore Chinese [21]. The effect of fermented soy products might differ from that of non-fermented soy foods because fermented soy products contain nitrosamines and nitrosamine precursors, which are known to be potential carcinogens [22]. Therefore, the fermentation process might mask the protective effect of non-preserved soy products observed in previous studies. Overall, neither the results from previous studies nor our findings in this study suggest a beneficial effect of soy foods in NPC.

The null association observed in this study was unlikely to have been due to a limited sample size. We had over 80% power to detect an OR≤ 0.725 (or ≥ 1.300) at a significance level of 0.05. Consistent with another report by Zhang et al. [23] on the intake of soy products in adult females conducted in the same region, our study population reported much lower consumption of isoflavones (mean intake of 7.22 mg/d) than that of Shanghai female residents (30.8 mg/d in Shanghai Women’s Health Study during 1997–2005) [8]. In the Shanghai study, consumption of soy foods significantly reduced lung cancer risk in nonsmoking women. In Zhang et al’s study [23], there was a significant inverse association between soy intake and breast cancer in women with similar low intake. These findings raised the question as to whether the lack of association between soy products and NPC was caused by low intake or was specific to different cancers. In our study, an inverse association was suggested between the intake of intermediate levels of total isoflavones and NPC risk in males and early TNM stage. However, this inverse relationship might have been caused by chance due to the multiple tests in our analyses.

In vivo studies and animal research have found the effects of soy products on NPC to be ambiguous. Soy differs from other legumes in that it possesses chemicals that are structurally and biologically similar to estrogen. It has been hypothesized that the estrogen receptor (ER) repressor, which is highly expressed in NPC tissue [24], can inhibit NPC cell growth and paradoxically promote the invasion of host tissues [25,26]. Hence, isoflavones were thought to play a dual role in NPC carcinogenesis by competing with estrogen in binding ER. Other constituents of soy products, such as, folate [27] and calcium [28], might account for the protective effect on carcinogenesis. Therefore, we suspect that the lack of association between soy products and NPC risk in our study is caused by conflicting effects of soy products on NPC.

To the best of our knowledge, this is the first and the largest study to investigate the association between NPC risk and soy proteins and isoflavones. We used a structured questionnaire with a comprehensive list of all the soy products commonly consumed in Guangdong, enhancing the chances to adequately reflect the spectrum of variation among the study subjects (correlation coefficient for soy was 0.45) [14]. Our study population was suited for an epidemiological investigation of soy products as it had a higher intake of soy compared with its western counterparts (averaging <0.5 mg/d for total isoflavones) [29]. In addition, the matching study design and full-scale adjustment for potential confounders facilitated the assessment of the effect of soy proteins and isoflavones on NPC in our population. Prevalence-incidence bias was also controlled for by limiting the recruitment of patients to those diagnosed within the previous 3 months.

However, this study had several limitations. First, we could not exclude the possibility that null association of soy and NPC was due to the relatively low intake. However, the participants of the highest quartile consumed ~20g/d (in soy). That is not a low dose for a habitual intake. Although many interventional studies supplemented with very large doses of soy foods or soy protein, it is unpractical to be generalized to a common population. Next, non-differential misclassification of soy intake based on FFQ due to the great random error in the soy food assessment would attenuate any true associations, although this questionnaire was validated in our source population [14]. Third, hospital-based studies tend to be subject to selection bias. This type of error was minimized by recruiting controls from a comparable study period (within 6 months) and from a hospital with a similar catchment population to the cases. We did not collect community-based controls due to limited resources to recruit controls in the communities among south China, and thus could not exclude the potential Berkson’s bias. Fourth, we couldn’t exclude interviewer bias because interviewers were not blinded to case/control status. However, we minimized interviewer biases by employing well-trained and experienced staff who interviewed equal proportions of cases and controls.Fifth, it was not sufficient to collect dietary information for only one year preceding the interviews. Any changes in dietary habits might have masked the true effects due to long duration of dietary exposure before the onset of NPC. Nevertheless, from longitudinal studies [30], we observed no essential changes in dietary habits of the adults. Exclusion criteria were also used during recruitment to confine subjects to those reporting a relatively stable dietary habit over the previous five years, so dietary changes should not have greatly biased the associations in our study. Last, it is difficult for us to analysis to control the influence of EBV because almost all the NPC cases were EBV seropositive (EBV VCA-IgA, 93%) and we did not examine the EBV status in the control.

In conclusion, our findings do not support the hypothesis that greater habitual consumption of soy products may be beneficial for preventing NPC in south Chinese adults with a relatively intake. Because of the relatively lower intake of soy products in the study population, further studies are needed to clarify this question in populations with a higher intake of soy products.
